# Supplementary material for: MetaRibo-Seq measures translation in microbiomes
Source: Nat Commun. 2020 Jun 29;11:3268. doi: 10.1038/s41467-020-17081-z (PMC7324362; doi:10.1038/s41467-020-17081-z)
Supplement: Supplementary file 10 — Supplementary Data 7 [file 41467_2020_17081_MOESM10_ESM.zip › File2/Confidence_VeryHigh_Taxonomy/377930_out.krona.html]

Javascript must be enabled to view this page.

members
magnitude
magnitudeUnassigned
count
unassigned
taxon
rank

377930\_out

59

59
2
superkingdom

976
phylum
59

59
200643
class

59
171549
order

815
family
1

816
genus
1

820
species
1

SRS078419\_contig\_number\_19754

171552
family
58

58
838
genus


SRS012916\_contig\_number\_3715SRS1041134\_contig\_number\_13559SRS143181\_contig\_number\_172SRS143876\_contig\_number\_3255SRS144506\_contig\_number\_42819
5
species
59823

1263102
species
1

SRS144537\_contig\_number\_35569

165179
species
52

SRS012969\_contig\_number\_2123SRS012989\_contig\_number\_7179SRS013521\_contig\_number\_390SRS013687\_contig\_number\_23515SRS013687\_contig\_number\_32563SRS014415\_contig\_number\_1778SRS015590\_contig\_number\_contig-100\_13390.54461SRS015782\_contig\_number\_37367SRS015782\_contig\_number\_41401SRS018888\_contig\_number\_2591SRS018888\_contig\_number\_9271SRS018888\_contig\_number\_contig-100\_1735.1736SRS019285\_contig\_number\_56SRS019910\_contig\_number\_18971SRS019910\_contig\_number\_contig-100\_169.140443SRS022609\_contig\_number\_5411SRS023914\_contig\_number\_26423SRS024132\_contig\_number\_24944SRS043841\_contig\_number\_9224SRS044535\_contig\_number\_13652SRS046502\_contig\_number\_12834SRS047044\_contig\_number\_13759SRS047433\_contig\_number\_10584SRS049712\_contig\_number\_20259SRS049896\_contig\_number\_19538SRS049995\_contig\_number\_5079SRS053398\_contig\_number\_17108SRS065397\_contig\_number\_4312SRS074586\_contig\_number\_5210SRS077194\_contig\_number\_908SRS077641\_contig\_number\_3094SRS077849\_contig\_number\_23165SRS078176\_contig\_number\_10999SRS078419\_contig\_number\_30685SRS097958\_contig\_number\_10916SRS097958\_contig\_number\_16695SRS103987\_contig\_number\_962SRS104036\_contig\_number\_6441SRS1041116\_contig\_number\_contig-100\_263.56524SRS104327\_contig\_number\_4808SRS104327\_contig\_number\_contig-100\_1539.44072SRS105082\_contig\_number\_648SRS143181\_contig\_number\_234SRS143181\_contig\_number\_528SRS144297\_contig\_number\_8847SRS144297\_contig\_number\_13733SRS144603\_contig\_number\_14234SRS144714\_contig\_number\_14859SRS144714\_contig\_number\_15885SRS146764\_contig\_number\_5218SRS146764\_contig\_number\_17770SRS148253\_contig\_number\_contig-100\_548.96956
